# Chemical stress drives opposing bacterial biomass responses at the single-cell level

**DOI:** 10.1093/ismeco/ycag281

**Published:** 2026-10-08

**Authors:** Saskia Rughöft, Samuel Zargani, Emanuel G Armanu, Dieter A Baumgartner, Lars Behrendt

**Affiliations:** Department of Organismal Biology, Uppsala University, Norbyvägen 18A, SE-752 36 Uppsala, Sweden; Department of Organismal Biology, Uppsala University, Norbyvägen 18A, SE-752 36 Uppsala, Sweden; Department of Chemical and Biochemical Engineering, Western University, Thompson Engineering Building, London, ON N6A 5B9, Canada; Institute of Environmental Engineering, Department of Civil, Environmental and Geomatic Engineering, ETH Zurich, 8049 Zürich, Switzerland; Division of Geological and Planetary Sciences, California Institute of Technology, Pasadena, CA 91125, United States; Department of Organismal Biology, Uppsala University, Norbyvägen 18A, SE-752 36 Uppsala, Sweden; Department of Biotechnology and Biomedicine, Technical University of Denmark, Søltofts plads 221, DK-2800 Kgs. Lyngby, Denmark

**Keywords:** microbial biomass, single-cell physiology, trait-based ecology, cellular heterogeneity, microbial stress responses, aquatic microbial ecology, nutrient cycling

## Abstract

Microbial biomass is a central functional trait governing nutrient turnover and trophic transfer in aquatic ecosystems, yet how chemical stress reshapes biomass at the level of individual cells remains poorly resolved. Conventional toxicity assays primarily detect population-level growth inhibition and overlook sublethal physiological responses and heterogeneity within microbial populations. Here, we quantify single-cell dry-mass, a label-free proxy for cellular biomass and biosynthetic state, in the fast-growing marine bacterium *Vibrio natriegens*. Across thousands of cells, we assessed how exposure to copper, zinc, diclofenac, bisphenol A, bisphenol E, and bisphenol Z alters cellular biomass. Chemical stress elicited contrasting responses: copper, zinc, and diclofenac reduced median dry-mass by up to 36%, whereas bisphenols increased median dry-mass by up to 24%. These changes were not simply proportional to population-level growth inhibition. Beyond shifts in central tendency, stress altered the shape and variability of biomass distributions, revealing additional distribution-level responses that are not detectable in bulk measurements. These results identify single-cell dry-mass as an integrative trait of microbial stress physiology and show that chemical perturbations can drive opposing cellular biomass responses that are not fully captured by population growth measurements. By linking chemical stress to microbial biomass traits, this work provides a controlled framework for testing cellular-scale responses in more complex ecological settings.

## Graphical Abstract

**Graphical Abstract:**
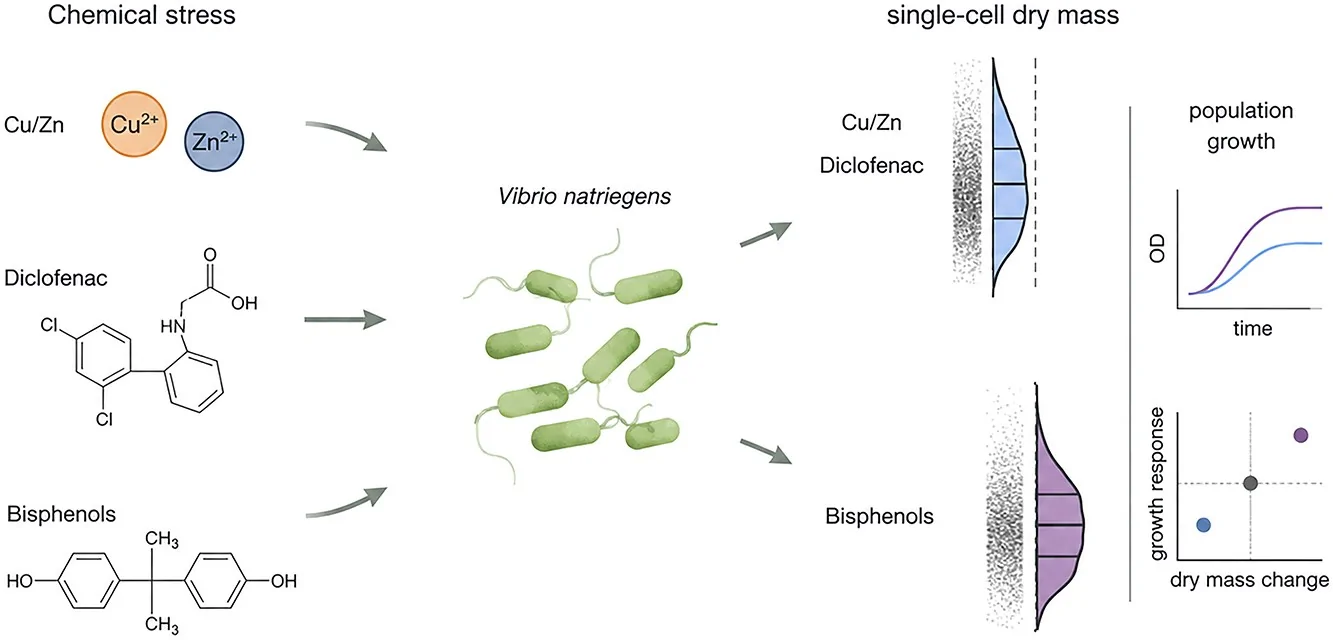

## Introduction

Microorganisms drive the transformation of matter and energy in aquatic ecosystems, linking primary production to nutrient remineralization through the microbial loop [1, 2]. Because their activity underpins ecosystem stability, even subtle physiological perturbations can propagate through food webs and alter biogeochemical fluxes. A central but poorly resolved aspect of this sensitivity is how microbial biomass, a key trait governing metabolic capacity and nutrient demand, responds to chemical stress at the level of individual cells.

Aquatic environments receive a growing diversity of chemical pollutants from human activities, generating ecological, economic, and public-health concerns [3–6]. Among the most widespread are heavy metals such as copper (Cu), zinc (Zn), manganese, cadmium, nickel, and iron [3, 7]. While Cu and Zn are essential micronutrients, they become toxic at elevated concentrations [8, 9]. Surveys reveal large variations in Cu and Zn across aquatic systems, from trace levels in pristine sediments to milligram-per-liter concentrations in contaminated sites [10–14]. Although the World Health Organization provides guideline values for Cu and Zn in drinking water [15], concentrations in polluted sediments and porewaters often far exceed these limits [7, 11–13, 16].

Pharmaceutical pollutants, introduced mainly via wastewater effluents, constitute another major class of aquatic contaminants. They are bioactive, persistent, and difficult to remove during treatment [17, 18]. The nonsteroidal anti-inflammatory drug diclofenac is among the most frequently detected compounds, occurring at microgram-per-liter concentrations in surface waters and sediments [19] and exerting toxic effects on diverse aquatic organisms [19–21]. Owing to its prevalence, diclofenac was added to a European Watch List [22]. Bisphenol A (BPA), a common plasticizer with endocrine-disrupting properties, is similarly ubiquitous, occurring in water and sediments at nano- to microgram-per-liter concentrations [23–25]. In response to restrictions on BPA, structurally similar analogues such as bisphenol E (BPE) and bisphenol Z (BPZ) are increasingly produced and detected in the environment [25, 26], although their toxicological profiles remain incompletely characterized [27]. Rather than focusing on compound-specific toxicity, these chemically diverse stressors provide a means to probe how different classes of environmental perturbations alter microbial biomass physiology.

Despite their prevalence, microbial responses to such chemical stressors remain underexplored. Most toxicity assays rely on vertebrate models because of their relevance to human health, leaving microorganisms—key agents of carbon and nutrient recycling [1, 2]—underrepresented in ecotoxicological research. The bioluminescent marine bacterium *Aliivibrio fischeri* is one of the few microbial models used in standardized assays, where bioluminescence inhibition provides a rapid proxy for population-level toxicity [28–30]. Members of the genus *Vibrio* are particularly important heterotrophs in marine and estuarine environments owing to their metabolic versatility and rapid responses to environmental change [31–33]. Among them, *Vibrio natriegens* has emerged as both an environmental and biotechnological model organism because of its exceptionally short doubling time—often under 10 min in optimal conditions [34–37]. This physiological speed enables rapid adjustment of cellular biomass and biosynthetic state, making *V. natriegens* a suitable system for resolving rapid trait-level adjustments in microbial biomass that may precede detectable growth inhibition.

However, despite the availability of fast-growing and ecologically relevant microbial model systems, most microbial toxicity tests still measure only bulk growth inhibition and overlook physiological heterogeneity among individual cells. Single-cell analyses can capture sublethal effects—such as altered biosynthetic balance or metabolic slowdown—that precede population-level growth inhibition. To quantify these changes directly, we employed quantitative phase imaging, a label-free optical technique that measures the optical-path difference of transparent cells, reflecting their refractive properties and dry-mass [38–40]. Because dry-mass represents the sum of all nonaqueous cellular material, it serves as a quantitative indicator of biosynthetic activity and total biomass [41, 42]. Quantitative phase imaging has been widely applied to eukaryotic systems [39, 43] and, more recently, to bacteria [44–49], revealing that dry-mass and density respond sensitively to environmental stress [46–48].

Here, we combine the rapid growth and ecological relevance of *V. natriegens* with automated, high-throughput quantitative phase imaging to quantify how chemical stress alters bacterial biomass at the single-cell level. Using exposure to Cu, Zn, diclofenac, BPA, BPE, and BPZ as defined perturbations, we assess both shifts in central tendency and changes in the structure of single-cell biomass distributions. By integrating single-cell biophysical measurements with a representative marine bacterium, this study establishes a quantitative framework linking chemical stress to cellular-scale variation in microbial biomass. We further test whether changes in cellular biomass track the corresponding population-level growth response. This framework provides a basis for testing how biomass-distribution traits respond to defined perturbations and may later be extended to mixed stressors, additional taxa, and natural microbial communities.

## Materials and methods

### Bacterial strain and pollutants

The type strain of *V. natriegens* (DSM 759) was obtained from the German Collection of Microorganisms and Cell Cultures (DSMZ). Copper sulfate (CuSO₄) and diclofenac sodium salt were purchased from Sigma-Aldrich (now Merck KGaA, Darmstadt, Germany). Ampicillin, paraformaldehyde (32%, methanol-free), and zinc sulfate (ZnSO₄·7H₂O) were purchased from VWR (Kista, Sweden). BPA, BPE, and BPZ were obtained from Chiron AS (Trondheim, Norway) as reference-grade compounds within the Partnership for the Assessment of Risks from Chemicals (PARC) project. Calibration beads (Thermo Scientific™ 4000 Series Monosized Particles) were purchased from Thermo Fisher Scientific (Göteborg, Sweden). These chemically distinct compounds were selected to represent different classes of environmental chemical stress. Stock solutions of pollutants were prepared using Milli-Q water or Dimethyl sulfoxide (DMSO) as indicated, stored at 4°C, and used within 1 week.

### Culture conditions and experimental setup

*Vibrio natriegens* was stored at −80°C in glycerol stocks and streaked on Luria-Bertani (LB) agar plates (1% w/v NaCl) for cultivation. Plates were incubated at 37°C overnight and maintained at room temperature for ≤3 weeks. For each experiment, an overnight culture was inoculated from a single colony and grown aerobically at 37°C and 220 rpm in LB medium supplemented with 1% NaCl. Exposure assays (Fig. 1) were performed in sterile 96-well tissue culture plates (VWR; Radnor, USA) using 10 μl of overnight culture per 200 μl of medium containing the pollutant. Exposure concentrations were chosen to span growth-supporting to growth-inhibitory regimes under the assay conditions, based on ranges previously determined to affect growth and to fall within environmentally relevant levels (Table S1). Stock solutions of Cu, Zn, and diclofenac were prepared in water; BPA, BPE, and BPZ were prepared in DMSO. Final exposure concentrations were: Cu, Zn, diclofenac: 0.01, 0.1, 1, 10, 50, 100 mg L^−1^ and BPA, BPE, BPZ: 0.001, 0.01, 0.1, 1, 10 mg L^−1^. Controls included: (i) untreated cultures, (ii) DMSO carrier control (4 μl DMSO added), and (iii) 20 mg L^−1^ ampicillin as a negative control. All concentrations represent nominal stressor levels rather than compound-specific toxicity thresholds**.**

**Figure 1:**
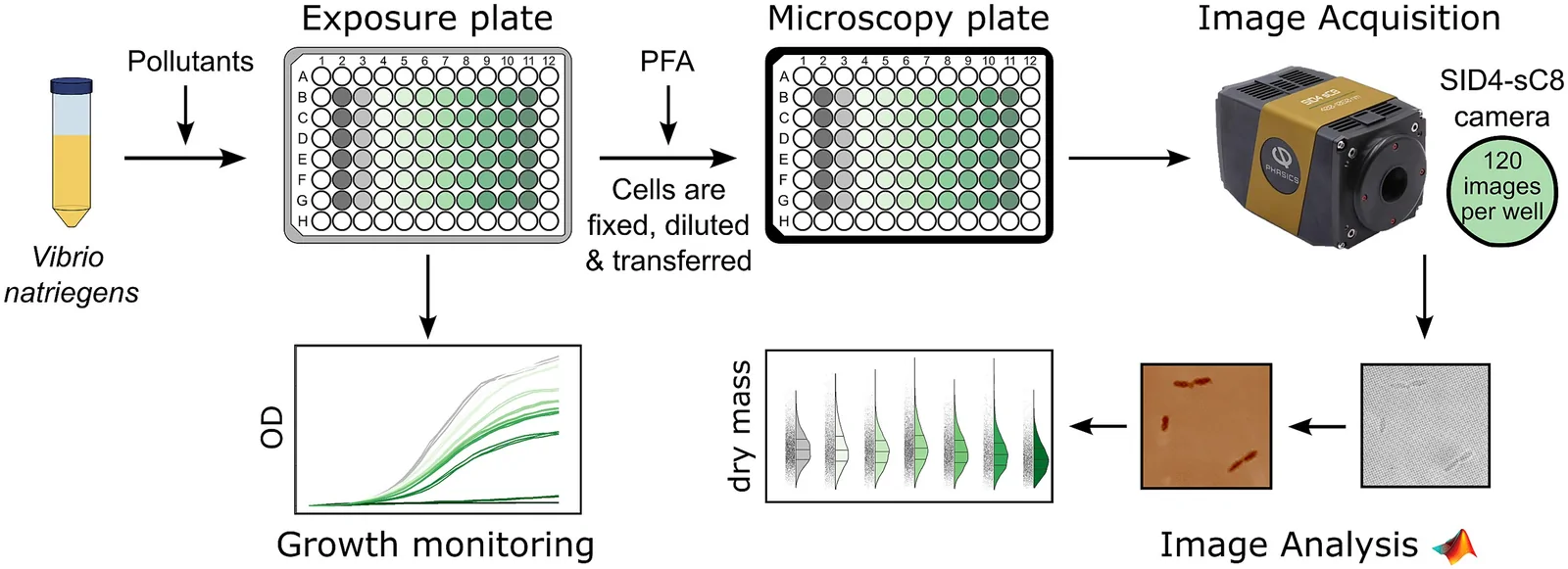
Experimental framework linking defined chemical stress to single-cell biomass measurements. Cultures of the marine bacterium *V. natriegens* were exposed to concentration gradients of selected chemical stressors in 96-well plates, while growth was monitored by optical density. After sampling at a defined physiological stage, cells were fixed with paraformaldehyde, diluted, and transferred to glass-bottom microscopy plates for single-cell imaging by quantitative phase imaging. Interferometric images were processed using a custom analysis pipeline to quantify dry-mass for thousands of individual cells per treatment. Representative phase and bright-field images are shown. SID4-sC8 QPI camera image reproduced with permission from Phasics.

Plates were incubated at 37°C in a Spark multimode reader (Tecan; Männedorf, Switzerland) with shaking (180 rpm) and optical density (OD_6_₀₀) recorded every 5 min after 10 s of mixing. Each experiment included four biological replicates (independent overnight cultures from different colonies) with two technical replicates per treatment. Cultures were sampled at the end of the initial rapid growth phase, determined independently for each exposure experiment from the matched untreated control growth curve measured in the same plate. This point was operationally identified as the onset of plateauing in the control OD_600_ trajectory, yielding an average sampling time of 3.4 ± 0.3 h, with individual sampling times ranging from 3.0 to 3.9 h (Fig. S1). This strategy was used to compare treatments at a comparable control-defined growth stage rather than at a fixed chronological time point.

Immediately after sampling, cells were fixed by adding paraformaldehyde to a final concentration of 3%, incubated for 15 min at room temperature, and then cooled to 4°C for storage until imaging. Cells were maintained in 3% paraformaldehyde and not washed prior to microscopic investigations. To monitor growth dynamics without pollutants, three replicate 24 h incubations were performed under identical conditions. Cultures were sampled at 1–8 h and 24 h by pausing incubation briefly to fix 200 μl aliquots as described above. These data were used to characterize how single-cell dry-mass changed across batch-growth phases and to identify the relevant sampling window for exposure experiments.

### High-throughput microscopy and quantitative phase imaging

Fixed cells were diluted in sterile LB medium to achieve a final 1:5–1:30 dilution. A 90 μl aliquot of diluted sample was added to a glass-bottom 96-well plate (Cellvis via IBL; Gerasdorf, Austria) and allowed to settle for ≥30 min, providing an estimated number of 80 000–100 000 cells at the bottom of each well for microscopy. Imaging was performed using an inverted microscope (Nikon Ti-2E Eclipse, Japan) equipped with a SID4-sC8 camera (Phasics; Saint-Aubin, France) and a 100× oil-immersion objective (CFI Plan Fluor, NA 1.30) with internal magnification for a final 150× total magnification. Automated image acquisition was conducted using NIS Elements JOBS routines, capturing 60 images per circular region (radius 0.5 mm, 1% overlap) at the center of each well; two nonoverlapping regions were imaged per well. Before imaging, a cell-free reference interferogram was recorded by gently moving the stage during a 2-s exposure at the same focal plane. Typical exposure times were 2–4 ms, and at least 3000 cells per replicate were analyzed.

### Image processing and dry-mass quantification

Image processing was performed using a custom MATLAB script (available at [50]). Interferogram and reference images were analyzed following algorithms from Baffou [40]. Background correction was applied to all phase images prior to segmentation. Cells were segmented using Otsu’s thresholding combined with noise filtering and watershed separation; parameters were optimized once and applied consistently across all datasets. Segmentation accuracy was visually validated for ≥10 fields per treatment. The image processing steps are summarized below. For all interferogram and reference images, intensity and wavefront profiles were extracted using the algorithms from Baffou [40], followed by a background correction step. Cells were segmented from the normalized wavefront/phase images using Otsu’s method thresholding, noise filtering, and watershedding. Segmentation parameters (e.g. cell size and circularity) were optimized for efficiency and applied consistently across all analyzed images. Finally, cell properties such as area, length, width, and dry-mass were calculated. Dry-mass values were calculated using the formula from Barer [38]:


$$ dm=\frac{1}{\alpha}\times area\times opd\times 0.8567 $$

where *dm* = dry-mass, *α* is the refractive increment, *area* = projected cell area and *opd* = average optical path difference (OPD) per cell. We used α = 0.18 ml g^−1^, a commonly applied refractive increment for cellular dry matter in interference microscopy and quantitative phase imaging [38]. This value converts phase-derived OPDs into dry-mass and represents an average for mixed cellular biomaterial. A correction factor of 0.8567, derived from calibration (see below), was applied to all measurements. Dry-mass density was calculated from cell volume assuming a spherocylindrical shape.

### Quantitative phase imaging calibration

Quantitative phase imaging calibration followed the protocol of Kwee *et al.* [51]. Polystyrene beads (2.5 μm diameter, refractive index = 1.59, *n* = 100) were imaged in immersion oil (refractive index = 1.518), and measured OPD values were compared to theoretical expectations. Polystyrene beads were used as a physical calibration standard, not as a biological analogue for bacterial material. Their defined diameter and refractive index allow theoretical OPD values to be calculated and compared with measured OPD values, thereby benchmarking the optical accuracy of the system. Measured OPD values of polystyrene beads were on average 16.7% (±3% SD) higher than expected, resulting in an empirical correction factor of 0.8567 applied to all dry-mass estimates. Bead diameters matched expected values within 2%, confirming spatial accuracy. Calibration results are shown in Fig. S2.

### Data analysis and availability

All data processing and visualization were performed in R v 4.3.2 [52] using the packages ggplot2, tidyverse, gghalves, and agricolae. Cell identification and region-of-interest (ROI) definition were performed during image segmentation: each accepted segmented object defined a single-cell ROI from which projected area, mean OPD, length, width, and dry-mass were extracted. Objects outside predefined size and shape criteria, as well as obvious OPD artifacts, debris, or merged cells, were excluded prior to analysis. For exposure experiments, four biological replicates were conducted per pollutant, each containing ≥3000 individual cells. Growth-monitoring experiments included three biological replicates. Statistical significance among treatments was evaluated using the nonparametric Kruskal–Wallis test followed by pairwise Wilcoxon tests (*P* < .05). Median dry-mass values are reported unless stated otherwise. Dose–response relationships were modeled using a quadratic function (*y* ~ *x* + *I(x^2^)*). All raw and processed data and plotting code are available from the corresponding author upon request. The single-cell dry-mass data supporting the main figures, representative imaging files, and the MATLAB script used for image processing are publicly available via Zenodo at [50].

### Genome annotation survey

To assess whether the available genome annotation of *V. natriegens* DSM 759 (= ATCC 14048) contained genes assigned to known bisphenol-degradation pathways, we examined the strain annotation in the JGI Integrated Microbial Genomes & Microbiomes (IMG/M) database (taxon ID 2811995373). KEGG pathway annotations were inspected using the IMG/M “Explore KEGG Modules, Pathways” function, focusing on the bisphenol degradation pathway (KEGG map00363) and adjacent aromatic-compound degradation pathways, including benzoate degradation (KEGG map00362). This survey was used only to assess the presence of annotated pathway components and was not intended as functional validation of bisphenol utilization.

## Results

### Single-cell dry-mass measurements reveal growth-dependent changes in *Vibrio natriegens*

Automated single-cell dry-mass measurements were used to quantify how growth phase and chemical stress influence microbial biomass in the fast-growing bacterium *V. natriegens* (Fig. 1). To distinguish pollutant-induced responses from natural variation during growth, we first characterized single-cell dry-mass dynamics over a 24 h incubation period in unperturbed cultures. Across three independent experiments (*n* = 69 364 cells), dry-mass values ranged from 0.1 to 1.8 pg, with a median of 0.39 pg [interquartile range (IQR) = 0.23]. Because single-cell dry-mass distributions deviated from normality, all central tendencies are reported as medians with IQRs, which represent the middle 50% of the data and provide a robust measure of spread for nonparametric datasets. Optical-density measurements revealed a biphasic growth pattern, with the first exponential phase ending after ~ 3 h (Fig. 2A). Median dry-mass increased by 25% during this phase, from 0.35 pg (IQR = 0.22, *n* = 5102) in stationary overnight cultures to 0.44 pg (IQR = 0.24, *n* = 7273) after 3 h (Fig. 2B). By 4 h, coinciding with the transition to a second growth phase, median dry-mass declined to 0.38 pg (IQR = 0.20). At the replicate level, the decrease in median dry mass from 3 to 4 h was statistically significant (Fig. 2B; Table S2). Variability peaked near 3 h (IQR = 0.24) and then decreased (IQR = 0.20) as cultures transitioned out of the initial growth phase (Fig. S3).

**Figure 2:**
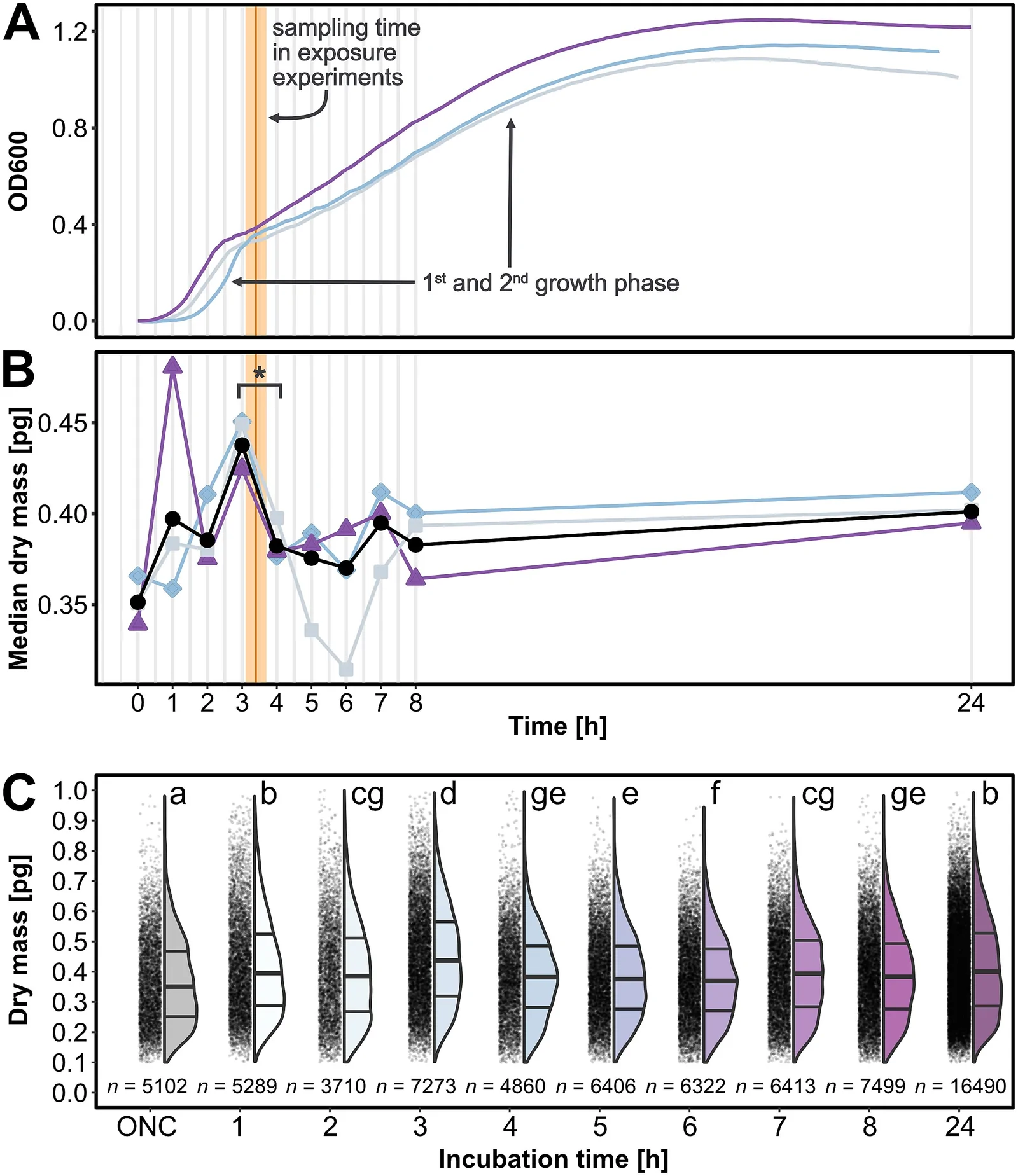
Growth-phase–dependent dynamics of single-cell dry-mass in *V. natriegens*. (A) Optical-density (OD_6_₀₀) growth curves of untreated cultures over 24 h (*n* = 3 biological replicates). The orange vertical marker indicates the approximate sampling window used for exposure experiments (3.4 h ± 0.3), which was determined independently for each exposure plate from matched untreated control growth curves. (B) Median single-cell dry-mass across the 24 h incubation period (*n* = 3). Lines represent individual replicates; and the black line denotes the pooled median. * denotes a statistically significant difference between values after 3 h and 4 h (Kruskal–Wallis test; *P* < .05; see Table S2). (C) Distributions of single-cell dry-mass in paraformaldehyde-fixed cultures sampled from overnight culture (ONC) and after 1–8 h and 24 h of incubation. Data are pooled from three independent experiments. Violin plots show distribution density with quartile lines (thicker line = median), overlaid with single-cell data points. Sample sizes (*n*) are indicated below each distribution. Different letters denote statistically significant differences among time points (Kruskal–Wallis test; *P* < .05).

Based on this growth-phase characterization, exposure experiments were sampled at experiment-specific, control-defined time points averaging 3.4 ± 0.3 h, with a range of 3.0–3.9 h (Fig. 2, Fig. S1), rather than at a fixed chronological time point. Growth curves recorded during the exposure experiments provided population-level context for interpreting the dry-mass measurements. Lower concentrations generally supported culture growth, whereas higher stressor concentrations caused growth inhibition for all compounds, with the strongest impairment at 10 mg L^−1^ of BPA, BPE, and BPZ or 50–100 mg L^−1^ for Cu, Zn, and diclofenac (Fig. S4).

### Chemical stress class determines the direction of biomass change

Exposure to chemically distinct stressors caused pronounced shifts in single-cell dry-mass that followed stress-class-associated patterns. Cu, Zn, and diclofenac exposure reduced cellular dry-mass, whereas bisphenol exposure increased it (Figs 3 and 4).

**Figure 3:**
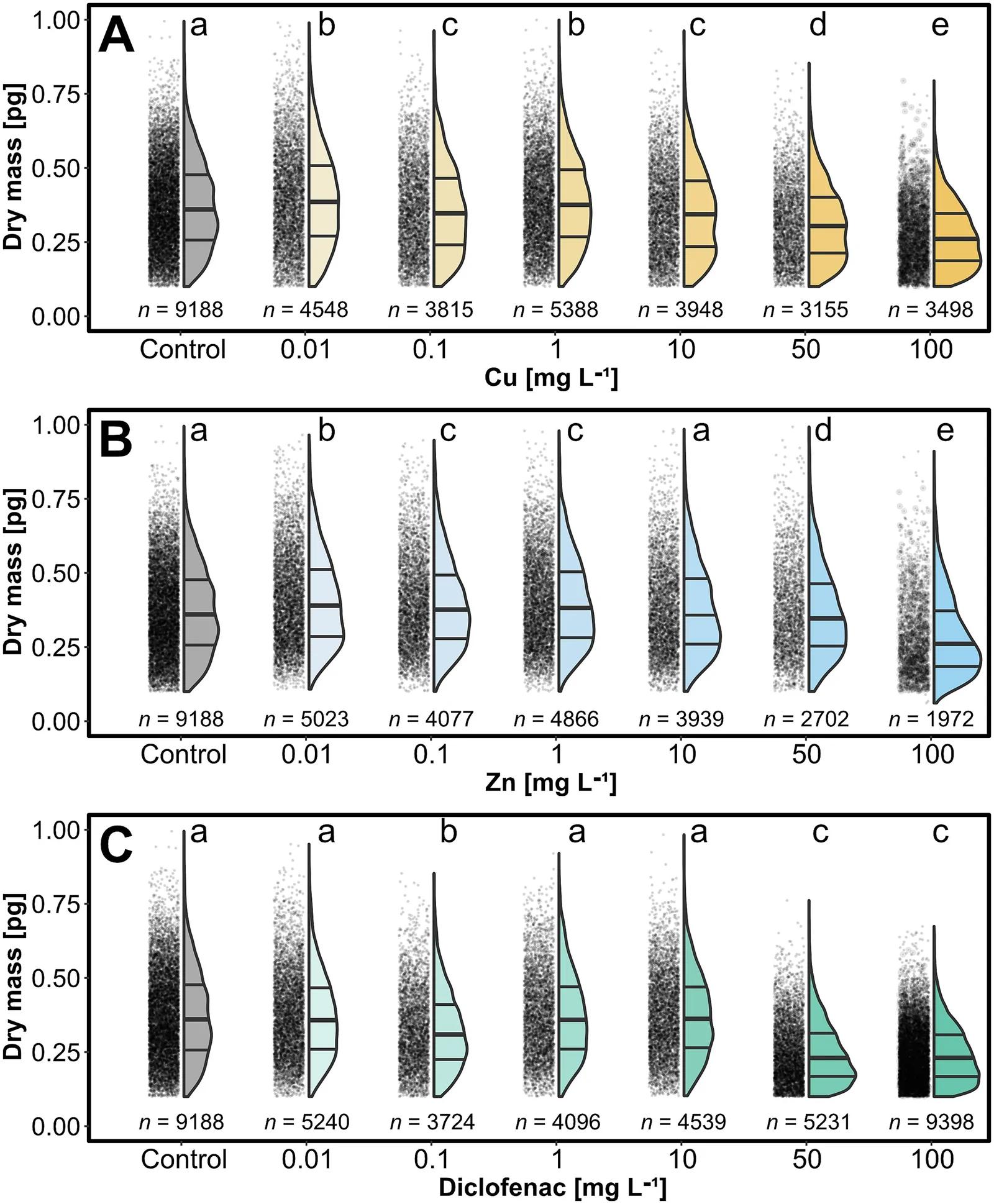
Chemical stress with metals and a pharmaceutical reduces single-cell dry-mass in *V. natriegens*. Distributions of single-cell dry-mass measured in paraformaldehyde-fixed cultures after 3.4 ± 0.3 h (Fig. S1) exposure to increasing concentrations of (A) Cu, (B) Zn, and (C) diclofenac. Data are pooled from four independent biological replicates. Violin plots show distribution density with quartile lines (thicker line = median), overlaid with single-cell data points. Sample sizes (*n*) are indicated below each distribution. Different letters denote statistically significant differences among treatments within each panel (Kruskal–Wallis test; *P* < .05).

**Figure 4:**
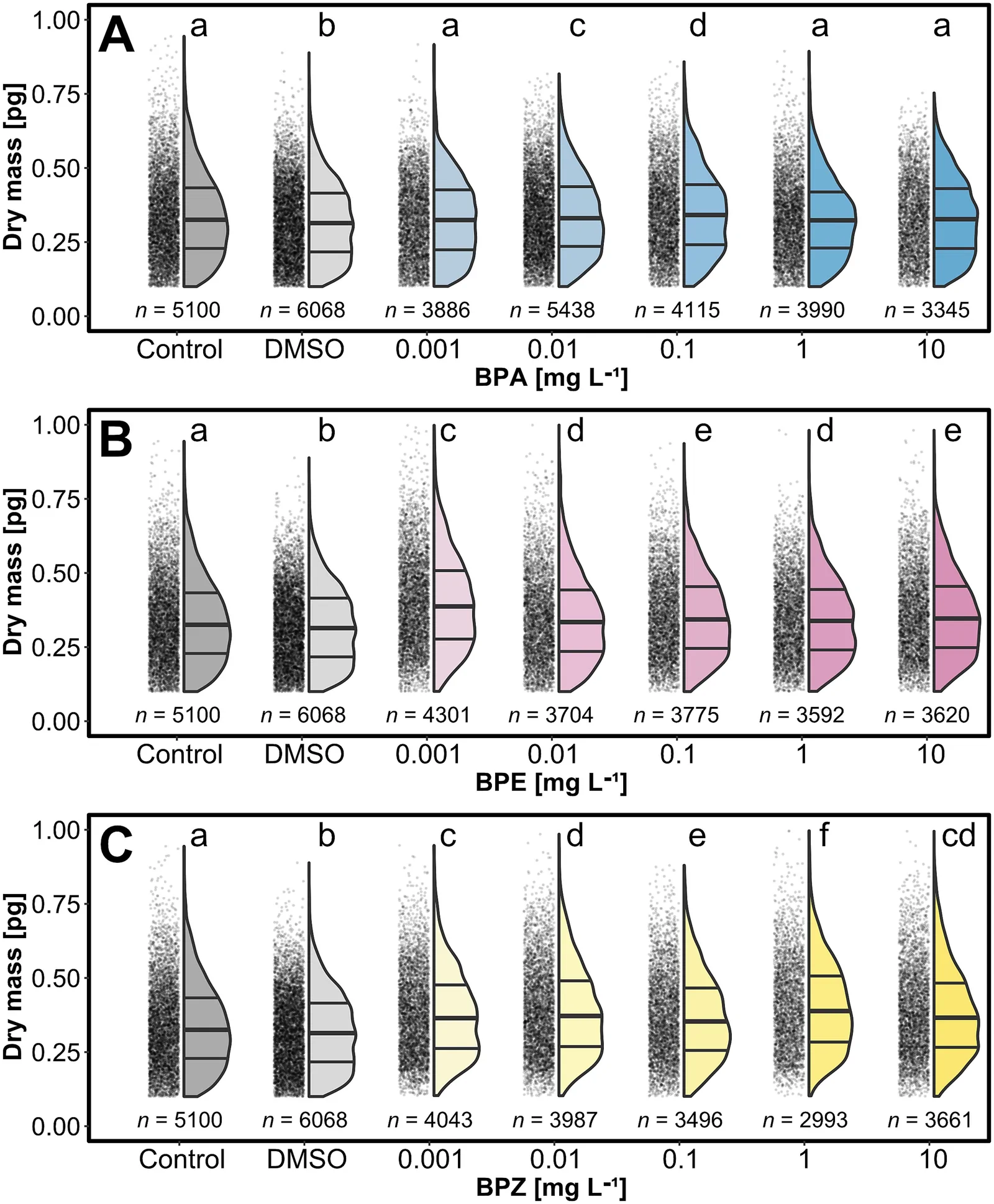
Concentration-dependent shifts in single-cell dry-mass under bisphenol stress in *V. natriegens*. Distributions of single-cell dry-mass measured in paraformaldehyde-fixed cultures after 3.4 ± 0.3 h exposure to increasing concentrations of (A) BPA, (B) BPE, and (C) BPZ, including untreated and DMSO controls. Data are pooled from four independent biological replicates. Violin plots show distribution density with quartile lines (thicker line = median), overlaid with single-cell data points. Sample sizes (*n*) are indicated below each distribution. Different letters denote statistically significant differences among treatments within each panel (Kruskal–Wallis test; *P* < .05).

#### Metals and pharmaceuticals

Exposure to Cu, Zn, and diclofenac led to concentration-dependent reductions in dry-mass (Fig. 3A-C; Table S3). The strongest decrease (by 36%) occurred in cultures exposed to 50 mg L^−1^ diclofenac, where the median dry-mass dropped from 0.36 pg in unexposed controls (IQR = 0.22, *n* = 9188) to 0.23 pg in exposed treatments (IQR = 0.15, *n* = 5231). Both Cu and Zn exposure at 100 mg L^−1^ also resulted in a strong decrease (by 28%) in the median dry-mass (Cu: control 0.36 pg → treatment 0.26 pg, IQR 0.22 → 0.16, *n* = 3498; Zn: 0.36 pg → 0.26 pg, IQR 0.22 → 0.19, *n* = 1972). At lower concentrations, responses were more variable: Zn at 0.01 mg L^−1^ increased median dry-mass by 9% (0.36 pg → 0.39 pg, *n* = 5240), whereas Cu exposure in the same range produced small but inconsistent shifts (−4 to +7%). Diclofenac exposure below 1 mg L^−1^ had minimal impact except at 0.1 mg L^−1^, where a 14% reduction was observed (0.36 pg → 0.31 pg, IQR 0.22 → 0.18, *n* = 3724). These effects were mirrored by size-normalized dry-mass density values (Figs S5 and S6), indicating that pollutant-induced decreases were not explained solely by changes in projected cell size, but reflected changes in cellular biomass density or composition.

#### Bisphenols

All bisphenol treatments produced higher median dry-mass values than DMSO controls (Fig. 4A–C; Table S4). DMSO alone slightly lowered median dry-mass by 3% relative to untreated controls (0.32 pg → 0.31 pg, IQR 0.21 → 0.20). BPA caused modest increases, with a maximum of +9% at 0.1 mg L^−1^ compared to DMSO controls (0.31 pg → 0.34 pg, IQR 0.20 → 0.20 pg, *n* = 4115 and 5100). BPE showed stronger effects, with a 24% rise at 0.001 mg L^−1^ (median = 0.39 pg, IQR = 0.23, *n* = 4301). Higher BPE concentrations (0.01–10 mg L^−1^) maintained elevated but stable dry-mass values. BPZ exposure increased dry-mass significantly at all concentrations tested, peaking at 1 mg L^−1^ (+24%; median = 0.39 pg, IQR = 0.22, *n* = 2993). This contrast indicates that while metal and pharmaceutical stress suppress biosynthetic capacity, bisphenol-associated stress favors biomass accumulation or retention even at sub-mg L^−1^ concentrations, an effect also mirrored by size-normalized dry-mass density values (Figs S6 and S7).

### Stress-induced changes in biomass distributions

Beyond changes in median biomass, pollutants also altered the shape and variability of dry-mass distributions within bacterial populations (Fig. 5B). For Cu, Zn, and diclofenac, the IQR decreased with increasing concentration—from 0.22 pg in controls to 0.14 pg at 100 mg L^−1^ diclofenac (Table S3)—indicating reduced heterogeneity among individual cells under stronger stress. This contraction suggests that, at high pollutant concentrations, the population converged toward a narrower range of biomass states. Population-level growth data show that these treatments still supported culture growth (Fig. S4), indicating that the narrowed dry-mass distributions were observed in cultures that had not undergone complete growth arrest. However, these bulk measurements cannot distinguish whether growth reflects broadly slowed division across the population or continued proliferation of only a subset of cells. Complementary mother-machine time-lapse observations for 100 mg L^−1^ diclofenac, a condition included in the dry-mass experiments, showed pronounced cell elongation and delayed division relative to untreated controls (Fig. S8). These single-cell observations therefore provide direct evidence of altered elongation and division dynamics under diclofenac stress, which cannot be resolved from bulk OD measurements alone. Because the mother-machine observations were qualitative and involved a limited number of tracked cells, they do not establish the fraction of actively dividing cells or the viability of cells in the Quantitative Phase Imaging (QPI) experiments. Together, the data support a conservative interpretation of the narrowed distributions as stress-associated convergence toward a restricted range of biomass states, rather than evidence for a specific resilient subpopulation.

**Figure 5:**
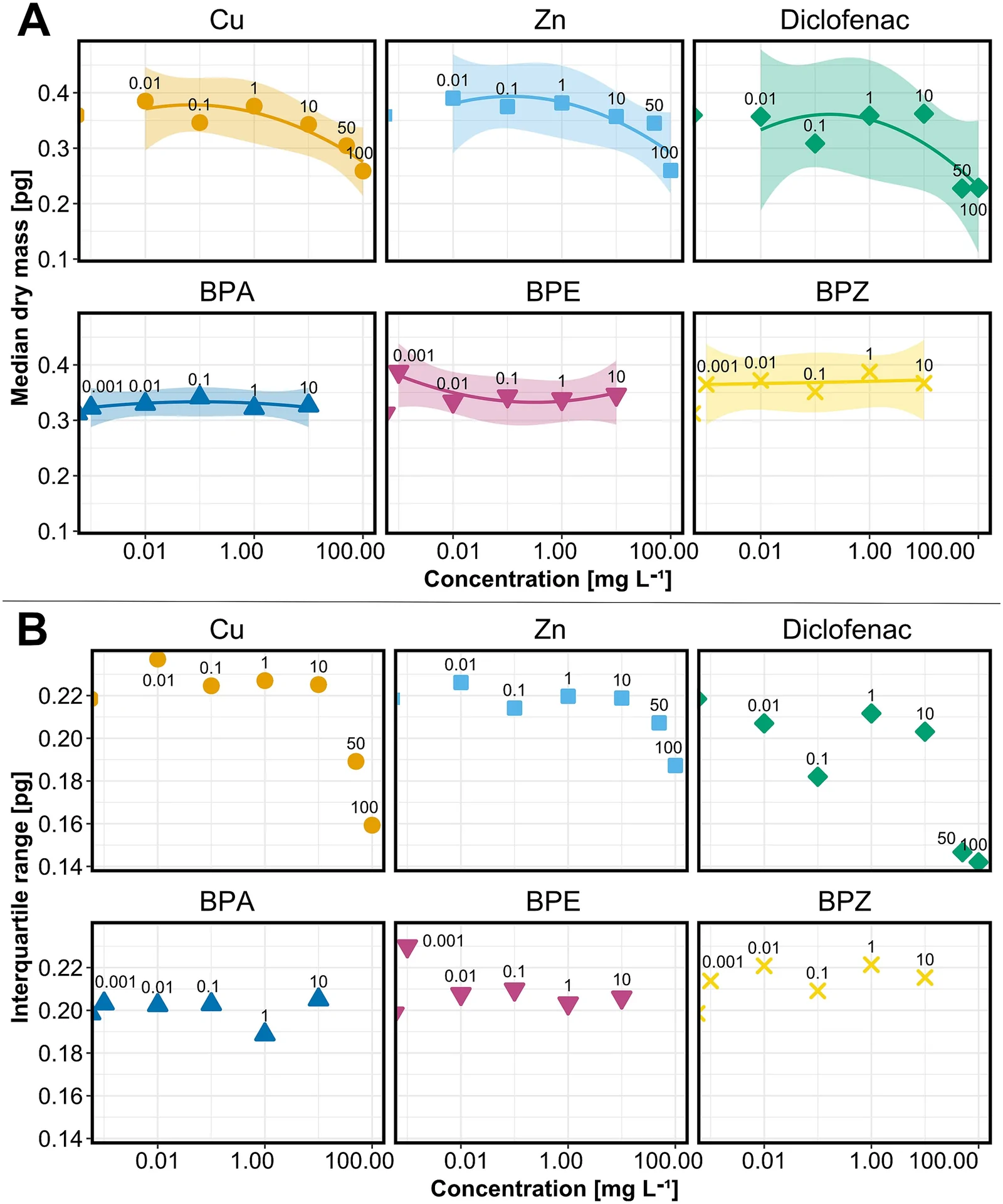
Summary statistics of single-cell dry-mass distributions under chemical stress in *V. natriegens*. (A) Median single-cell dry-mass across increasing concentrations of Cu, Zn, diclofenac, and bisphenols A, E, and Z. Solid lines represent quadratic fits (*y* ~ *x* + *I(x^2^)*) across log-scaled concentrations. (B) IQR of single-cell dry-mass across the same concentration gradients. Values are derived from the datasets shown in Figs 3 and 4.

For bisphenol exposures, variability showed no consistent trend across concentrations, but the symmetry of distributions shifted. Skewness values increased with concentration for most compounds (Fig. S9), reflecting a greater relative contribution of cells at the high-dry-mass end of the distribution. BPA, which had the weakest overall effect on median dry-mass, was the only compound that did not exhibit this increase in skewness. Together, these results demonstrate that chemical stress reshapes both the central tendency and the structure of microbial biomass distributions: metal and pharmaceutical stress compresses biomass states, whereas bisphenol stress broadens or skews them, producing more asymmetric biomass distributions. Single-cell quantitative phase imaging thus reveals distributional biomass responses that are not captured by bulk population measurements alone. To relate single-cell biomass responses to population-level growth, we mapped treatment effects into an empirical response space defined by relative changes in median dry-mass and growth inhibition calculated from maximum OD_600_ (Fig. 6A). This analysis showed that dry-mass responses were not simply proportional to growth inhibition. Cu, Zn, and diclofenac clustered primarily in a biomass-loss/growth-inhibition region, consistent with reduced cellular biomass under increasingly inhibitory stress. In contrast, bisphenol exposures shifted cells toward increased dry-mass while producing weaker or more variable growth inhibition (Fig. 6B). The response space therefore separates stressor classes that reduce both growth and cellular biomass from those that increase cellular biomass without equivalent suppression of population growth.

**Figure 6:**
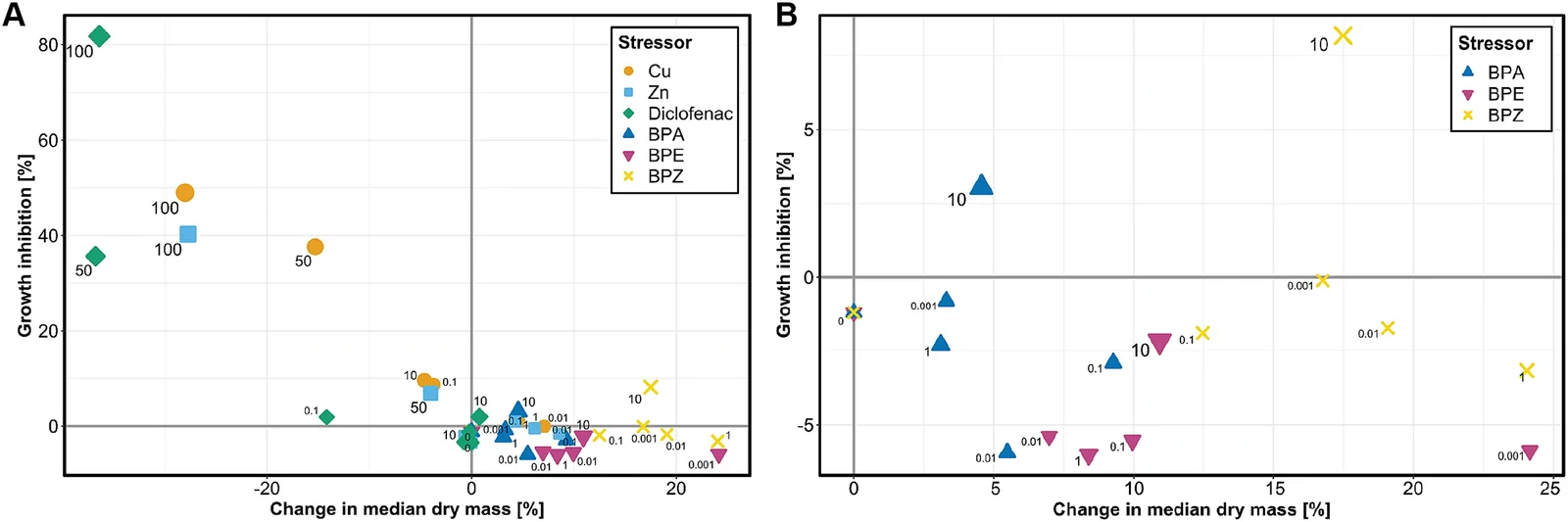
Relationship between population growth inhibition and single-cell dry-mass responses in *V. natriegens*. (A) Growth inhibition was calculated from maximum OD_600_ relative to the corresponding control, with positive values indicating reduced population growth and negative values indicating growth exceeding the control. Relative changes in median single-cell dry mass were calculated relative to the corresponding control, with negative values indicating reduced dry mass and positive values indicating increased dry mass. Points show responses across increasing concentrations of Cu, Zn, diclofenac, BPA, BPE, and BPZ. Exposure concentrations are indicated by plot labels in mg L^−1^ and marker size increases with concentration. (B) Enlarged view of the BPA, BPE, and BPZ responses shown in panel A, highlighting the shift toward increased cellular dry mass across bisphenol exposures despite weaker or more variable effects on population growth. The response space separates treatments associated with biomass loss and growth inhibition from treatments that increased dry mass with weaker effects on population growth.

## Discussion

Microorganisms regulate the cycling of matter and energy in aquatic ecosystems, yet quantitative descriptions of how chemical stress reshapes microbial biomass—a central functional trait underlying ecosystem metabolism—remain scarce. Here we link single-cell biophysics with defined chemical perturbations to reveal how chemical stress alters microbial biomass at the cellular level and how these responses relate to population growth. Using the fast-growing marine bacterium *V. natriegens* as a model, we show that distinct classes of chemical stress exert opposing effects on cellular dry-mass: metals (Cu, Zn) and a pharmaceutical (diclofenac) decreased biomass, whereas bisphenols (BPA, BPE, BPZ) increased it. Importantly, these changes in cellular biomass were not simply proportional to the corresponding effects on population growth. These patterns were consistent across biological replicates and mirrored by size-normalized dry-mass density measurements, supporting the interpretation that the observed changes reflect biomass-state responses rather than cell-size artifacts alone.

Before interpreting stress-induced effects, we validated the method by characterizing unexposed *V. natriegens* cultures. Most *V. natriegens* cells exhibited dry-mass values between 0.1 and 1 pg, consistent with reported measurements for this species [53], broader bacterial dry-matter relationships [54], and single-cell biomass of marine *Vibrio* isolates [55]. This confirms that the absolute values obtained by quantitative phase imaging are within the expected range for marine bacteria. The phase-dependent growth pattern observed here—an increase in dry-mass during exponential growth followed by a decrease as cultures entered stationary phase—corroborates earlier analyses of *V. natriegens* using X-ray microanalysis and electron microscopy [53] and of *Escherichia coli* using quantitative phase imaging [44]. Together, these comparisons support the use of quantitative phase imaging to capture both the absolute magnitude and temporal dynamics of cellular dry mass in bacteria. Importantly, cellular dry-mass is expected to vary with growth rate and physiological state. Classical bacterial growth-law studies showed that average cell size, cellular mass, and macromolecular composition scale with growth conditions, and later work linked these relationships to resource allocation and growth physiology [56, 57]. More recent single-cell mass measurements further show that batch cultures maintain stable mass distributions only over restricted physiological windows [58]. Thus, the dry-mass responses observed here should be interpreted as integrative shifts in growth state and biosynthetic allocation, rather than as compound-specific molecular mechanisms in isolation.

Although single-cell quantitative phase imaging studies of chemical toxicity remain relatively rare [39, 46, 47], the sensitivities observed here align with patterns reported from conventional assays. Microtox experiments using *A. fischeri* have shown greater sensitivity to Cu than Zn at sublethal concentrations [59, 60], consistent with our observation that *V. natriegens* responded more strongly to Cu under comparable exposure regimes. Reported ecotoxic effects for diclofenac [61, 62] and BPA in bioluminescent bacterial assays [63] similarly fall within the concentration ranges that altered dry-mass in our study. Previous work further indicates stronger toxicity of BPZ relative to BPA [25, 64], in agreement with the larger responses we detected, whereas BPE has been reported to exhibit higher half-maximal effect concentrations than BPA [64]. The partial discrepancy observed here, with BPE inducing slightly stronger biomass responses than BPA, underscores the need to resolve how structurally similar compounds differentially perturb microbial physiology.

Measuring dry-mass at the single-cell level also reveals how chemical stress reorganizes the distribution of biomass states within bacterial populations. While stress exposure often increases phenotypic heterogeneity in microbes [65, 66], we observed the opposite trend at high concentrations of heavy metals and diclofenac. Population-level growth curves showed that several treatments still supported culture expansion at the sampling time, although high concentrations caused marked growth inhibition. These data establish that narrowing of the dry-mass distributions was observed in cultures that had not undergone complete population-wide growth arrest. However, fixed dry-mass distributions cannot identify which individual cells were actively growing, growth-arrested, or nonviable, because survival and viability were not measured directly. Likewise, bulk growth measurements cannot distinguish broadly slowed growth across many cells from continued proliferation of only part of the population. Complementary mother-machine observations under diclofenac exposure provide qualitative evidence of altered single-cell elongation and division dynamics, but do not resolve the physiological state of the cells measured by QPI. We therefore interpret the narrowed distributions as stress-associated shifts in biomass state rather than evidence for a specific physiologically resilient subpopulation. Because bacterial cell mass is growth-rate dependent, this narrowing may reflect slower growth, reduced biosynthetic activity, or restriction of accessible biomass states rather than a distinct stress-induced reduction in phenotypic heterogeneity.

Together, these comparisons and distributional analyses show that quantitative phase imaging–based single-cell dry-mass measurements capture stress-associated biomass responses while revealing how chemical stress alters both the magnitude and structure of cellular biomass distributions. Importantly, the contribution of this study is not dry-mass measurement alone, but the demonstration that defined chemical stressors drive contrasting bacterial biomass responses at single-cell resolution. More broadly, the empirical response-space analysis shows that dry-mass responses are not simply proportional to growth inhibition. By relating changes in median dry-mass to growth inhibition, this framework separates biomass-loss/growth-inhibition responses, observed primarily for Cu, Zn, and diclofenac, from biomass-gain responses with weaker or more variable growth inhibition, observed for bisphenol exposures. Its practical value is therefore to add a cellular biomass dimension to conventional growth-inhibition measurements, allowing stressors with similar effects on population growth to be distinguished by their underlying biomass response and prioritized for mechanistic follow-up. We view this as a descriptive response map for interpreting biomass-response modes under controlled conditions, rather than as a predictive environmental model; testing whether such modes truly predict microbial functional capacity will require integration with survival measurements, mixed-stressor experiments, and validation across additional taxa and community contexts.

Cell dry-mass reflects the cumulative outcome of cellular composition, including macromolecular content, dry-mass density, and crowding state [67–70]. Previous studies have shown that nutrient availability, starvation, and antibiotic exposure can alter bacterial dry-mass, dry-mass density, and morphology [48, 49, 71]. Such changes may partly reflect shifts in macromolecular composition and cellular dry-mass density, both of which vary with physiological state [70, 72]. These principles suggest that chemical stressors could modify dry-mass by altering biosynthetic balance or macromolecular composition. In addition, stress-induced accumulation or depletion of intracellular storage compounds, such as polyphosphate, glycogen, or lipid inclusions, could alter refractive properties and contribute to OPD-derived dry-mass changes; these components were not directly quantified in the present study. For Cu in particular, toxicity has been linked to inhibition of nucleotide synthesis, protein aggregation, and membrane lipid peroxidation [73–75], mechanisms that would plausibly reduce biosynthetic capacity and thereby decrease cellular dry-mass. The contraction of dry-mass distributions observed at high Cu, Zn, and diclofenac concentrations is consistent with reduced biosynthetic activity or a restriction of accessible biomass states under acute stress. Since our data do not distinguish between active growth, growth arrest, or selective loss of sensitive cells, we interpret this pattern as a narrowed biomass-state distribution rather than direct evidence of metabolic persistence. In contrast, bisphenol exposure increased dry-mass, particularly for BPE and BPZ. Such increases could reflect either elevated macromolecular accumulation or the utilization of bisphenols as additional carbon sources, as degradation and co-metabolism of BPA and related compounds have been reported in other bacteria [76, 77]. However, to our knowledge, bisphenol degradation has not been documented for *V. natriegens*, and a survey of the *V. natriegens* DSM 759 genome annotation did not reveal a complete bisphenol-degradation pathway, although genes associated with downstream aromatic-compound metabolism, including benzoate degradation, were found. We did not measure bisphenol depletion during incubation and therefore cannot determine whether *V. natriegens* could have utilized these compounds as carbon sources under the conditions tested. Bisphenols can also affect membrane properties and cellular toxicity responses [78, 79], potentially leading to altered physiological state or stress-associated biomass accumulation. Although the precise mode of action remains unresolved, the directionality of these changes suggests that bisphenols, unlike metals or diclofenac, were associated with biomass accumulation or retention rather than consistent biomass loss.

The dry-mass of bacterial cells is ecologically relevant because it underpins microbial biomass estimates, nutrient demand, and the amount of cellular material available for recycling or grazing [1, 2, 70]. Stress-induced shifts in biomass distributions may therefore influence the size structure and allocation of microbial biomass within populations. By quantifying these shifts at single-cell resolution, our study provides a framework for measuring how chemical perturbations reshape microbial biomass traits. Determining whether such trait-level responses propagate to carbon and nutrient fluxes or can be used predictively in ecological and biogeochemical models will require mixed-stressor experiments in combination with phylogenetically diverse taxa or natural microbial communities.

## Supplementary Material

Supplementary_1_4_ycag281

## Data Availability

The datasets analyzed for the current study are available at Zenodo [50].
