# Supplementary material for: Chemical stress drives opposing bacterial biomass responses at the single-cell level
Source: ISME Commun. 2026 Oct 8;6(1):ycag281. doi: 10.1093/ismeco/ycag281 (PMC13685777; doi:10.1093/ismeco/ycag281)
Supplement: Supplementary file 1 [file supplementary_1_4_ycag281.docx]

**Chemical stress drives opposing bacterial biomass responses at the single-cell level**

*Saskia Rughöft^1^, Samuel Zargani^1^, Emanuel G. Armanu^2^,*

*Dieter A. Baumgartner^3,4^, Lars Behrendt^1,5^*^*^

^1^ Department of Organismal Biology, Uppsala University, Norbyvägen 18A, SE-752 36 Uppsala, Sweden

^2^ **Department of Chemical and Biochemical Engineering, Western University, Thompson Engineering Building, London, ON N6A 5B9, Canada**

^3^ Institute of Environmental Engineering, Department of Civil, Environmental and Geomatic Engineering, ETH Zurich, Zurich, Switzerland.

^4^ Division of Geological and Planetary Sciences, California Institute of Technology; CA 91125, USA

^5^ Department of Biotechnology and Biomedicine, Technical University of Denmark, Søltofts plads 221, DK-2800 Kgs. Lyngby, Denmark

*Corresponding author:*
Lars Behrendt
Department of Biotechnology and Biomedicine
Technical University of Denmark
Søltofts Plads 221
DK-2800 Kgs. Lyngby, Denmark
Email: labeh@dtu.dk

**SUPPLEMENTARY INFORMATION**

**Fig. S1: Growth curves of unexposed control cultures used to define experiment-specific sampling times in chemical stress experiments.** Only untreated control cultures are shown. Each control curve was measured on the same plate as the corresponding chemical-exposure series and was used to determine the sampling time for that independent experiment. Chemical-exposure growth curves are shown separately in Fig. S4. Sampling was performed at the end of the first growth phase, determined independently for each exposure experiment from the untreated control curve measured in the same plate and shown here. Sampling time points ranged from 3.0 to 3.9 h, with an average of 3.4 ± 0.3 h. Experiments I–IV denote four independent experiments for copper, zinc, and diclofenac exposure, each tested at 0.01, 0.1, 1, 10, 50, and 100 mg L⁻¹. Experiments V–VIII denote four independent experiments for bisphenol A, bisphenol E, and bisphenol Z exposure, each tested at 0.001, 0.01, 0.1, 1, and 10 mg L⁻¹.

**Alt text:** Untreated control growth curves from independent chemical-exposure experiments, showing the experiment-specific sampling points selected at the end of the first growth phase. Sampling occurred between 3.0 and 3.9 hours, with an average of 3.4 hours.

**Fig. S2: Calibration of quantitative phase imaging measurements.** Polystyrene beads (2.5 µm diameter, refractive index = 1.59; *n* = 100) were imaged in immersion oil (refractive index = 1.518), and measured optical path difference (OPD) values were compared to theoretical expectations. Mean OPD values were on average 16.7% (± 3% standard deviation) higher than expected, resulting in an empirical correction factor of 0.8567 applied to all dry-mass estimates. Measured bead diameters deviated by less than 2% from expected values, confirming spatial measurement accuracy.

**Alt text:** Calibration measurements comparing experimentally measured and theoretical optical path differences for 2.5-µm polystyrene beads. Measured optical path differences were approximately 16.7% higher than expected, while measured bead diameters were within 2% of their nominal size.

**Fig. S3: Dry-mass dynamics of *Vibrio natriegens* during biphasic growth.** (A) Interquartile ranges (IQR) of single-cell dry-mass distributions over a 24 h incubation period (*n* = 3 biological replicates). The black line represents IQR values calculated from pooled replicates.

**Alt text:** Interquartile ranges of single-cell dry-mass distributions during 24 hours of untreated *Vibrio natriegens* growth, shown for three biological replicates and for the pooled dataset, illustrating changes in population-level dry-mass variability across growth phases.

**Fig. S4: Representative growth curves of stressor-exposed cultures up until the sampling point for dry mass analysis.** Sampling was performed at the end of the first growth phase, determined independently for each exposure experiment from the untreated control curve measured in the same plate. Sampling time points ranged from 3.0 to 3.9 h, with an average of 3.4 ± 0.3 h. Exposure experiments included the stressors copper, zinc, and diclofenac exposure (each tested at 0.01, 0.1, 1, 10, 50, and 100 mg L⁻¹), as well as bisphenol A, bisphenol E, and bisphenol Z exposure (each tested at 0.001, 0.01, 0.1, 1, and 10 mg L⁻¹). Control treatments included unexposed controls, DMSO carrier controls for bisphenol exposure experiments and negative growth controls with 20 mg L^-1^ ampicillin. Growth data shown here represent one of four biological replicate experiments per stressor and average OD600 values of two technical replicates per treatment.

**Alt text:** Representative optical-density growth curves for *Vibrio natriegens* exposed to increasing concentrations of copper, zinc, diclofenac, BPA, BPE, or BPZ up to the dry-mass sampling point. Higher stressor concentrations generally produce stronger growth inhibition.

**Fig. S5: Increasing concentrations of heavy metals and diclofenac reduce the dry-mass density of *Vibrio natriegens*.** Single-cell dry mass was quantified after 3.4 ± 0.3 h of chemical stress with increasing concentrations of (A) copper, (B) zinc, and (C) diclofenac. Graphs include data from four independent biological replicates; total cell numbers are indicated for each treatment. Dry-mass density distributions are shown as half-violin plots with quartile lines (median indicated by a thicker line) overlaid with single-cell data points. Different letters denote statistically significant differences among treatments (Kruskal–Wallis test, *p* < 0.05).

**Alt text:** Single-cell dry-mass density distributions across increasing concentrations of copper, zinc, and diclofenac. High stressor concentrations shift the distributions toward lower dry-mass density, paralleling the decreases observed for cellular dry mass.

**Fig. S6: Summary statistics of single-cell dry-mass distributions in *Vibrio natriegens* under chemical stress**. Median dry-mass density values for cells exposed to copper, zinc, diclofenac, bisphenol A, bisphenol E, and bisphenol Z, with fitted quadratic dose–response curves (y ~ x + I(x²)) across log-transformed concentration gradients.

**Alt text:** Summary plots of median single-cell dry-mass density across concentration gradients of copper, zinc, diclofenac, BPA, BPE, and BPZ, with fitted quadratic curves showing the different concentration-dependent responses among stressors.

**Fig. S7: Bisphenol A and its structural analogues bisphenol E and bisphenol Z increase the dry-mass density of *Vibrio natriegens*.** Single-cell dry mass was quantified after 3.4 ± 0.3 h of chemical stress with increasing concentrations of (A) bisphenol A, (B) bisphenol E, and (C) bisphenol Z. Graphs include data from four independent biological replicates; total cell numbers are indicated for each treatment. Dry-mass density distributions are shown as half-violin plots with quartile lines (median indicated by a thicker line) overlaid with single-cell data points. Different letters denote statistically significant differences among treatments (Kruskal–Wallis test, *p* < 0.05).

**Alt text:** Single-cell dry-mass density distributions for increasing concentrations of BPA, BPE, and BPZ. Bisphenol exposure generally shifts dry-mass density toward higher values relative to the DMSO control.

**Fig. S8: Complementary mother-machine observations under chemical stress.** Representative mother-machine time-lapse images and single-cell length trajectories of *Vibrio natriegens* under untreated control conditions and diclofenac exposure (100 mg L⁻¹) over 45 min. Red boxes indicate representative tracked cells in microchannels. Control cells elongated and divided over the observation window, whereas diclofenac-exposed cells showed pronounced elongation with delayed or absent division. Each trajectory plot shows 10 tracked cells. These observations provide qualitative context for stress-associated changes in single-cell growth dynamics and morphology, but were not used to infer viability, dry mass, or the fraction of growing cells in the quantitative phase-imaging experiments.

**Alt text:** Mother-machine time-lapse images and cell-length trajectories comparing untreated cells with cells exposed to 100 mg L⁻¹ diclofenac. Control cells elongate and divide, whereas diclofenac-exposed cells show pronounced elongation with delayed or absent division during the observation period.

**Fig. S9: Summary statistics of single-cell dry-mass distributions in *Vibrio natriegens* under chemical stress**. Skewness of dry-mass distributions for cells exposed to copper, zinc, diclofenac, bisphenol A, bisphenol E, and bisphenol Z across log-transformed concentration gradients.

**Alt text:** Summary of skewness values for single-cell dry-mass distributions across concentration gradients of copper, zinc, diclofenac, BPA, BPE, and BPZ, showing how distribution asymmetry changes with chemical exposure.

**Table S1: Reported environmental concentration ranges of copper, zinc, diclofenac, bisphenol A (BPA), bisphenol E (BPE), and bisphenol Z (BPZ) in aquatic systems.** Concentration ranges are compiled from previously published studies and represent reported values in surface waters, sediments, or porewaters. Values are provided to contextualize the exposure concentrations used in this study.

| **Pollutant** | **Location** | **Sample material** | **Concentrations** | **Reference** |
| --- | --- | --- | --- | --- |
| Copper | marinas | water | 1.5 – 5.75 µg L^-1^ | [1] |
| Copper | Bohai Sea (China) | sediment porewater | 0.2 – 0.5 mg L^-1^ | [2] |
| Copper | USA | sediment porewater | 0.2 µg l^-1^ – 6 mg L^-1^ | [3] |
| Copper | ports & estuaries (worldwide) | sediment | 0.7 – 1060 mg kg^-1^ | [4] |
| Copper | global lakes & rivers | sediment | 27 – 268 mg kg^-1^ | [5] |
|  |  |  |  |  |
| Zinc | marinas | water | 2.6 – 11.8 µg L^-1^ | [1] |
| Zinc | Chinese estuary | porewater | 100 – 400 µg L^-1^ | [6] |
| Zinc | USA | sediment porewater | 0.5 µg l^-1^ – 27 mg L^-1^ | [3] |
| Zinc | Global lakes & rivers | sediment | 117 – 1012 mg kg^-1^ | [5] |
| Zinc | ports & estuaries (worldwide) | sediment | 4.2 – 14600 mg kg^-1^ | [4] |
|  |  |  |  |  |
| Diclofenac | worldwide | surface water | 6.2 ng l^-1^ – 57 µg L^-1^ | [7] |
| Diclofenac |  | sediment | up to 309 ng kg^-1^ | [7] |
| Diclofenac | worldwide | marine, coastal & estuarine water | up to 15 µg L^-1^ | [8] |
|  |  |  |  |  |
| BPA | Asian lakes & rivers | water | 1 – 678 ng L^-1^ | [9] |
| BPA | Europe | sediment | up to 190 ng g^-1^ | [10] |
| BPA | Europe | water | up to 0.8 µg L^-1^ | [11] |
|  |  |  |  |  |
| BPE | Europe | water | up to 2.77 µg L^-1^ | [12] |
|  |  |  |  |  |
| BPZ | Asian lakes & rivers | water | 0.05 – 45 ng L^-1^ | [9] |

**Table S2: Summary statistics of single-cell dry-mass distributions in unexposed *Vibrio natriegens* cultures over 24 h of growth**. Reported metrics include mean, median, interquartile range (IQR), coefficient of variation (CV), and percentage change in median dry mass. ONC denotes overnight culture. Different letters behind median values denote statistically significant differences among time points (Kruskal–Wallis test; *p* < 0.05).

| **Timepoint** | **Mean** | **Median** | | **IQR** | **CV** | **median change vs. ONC** |
| --- | --- | --- | --- | --- | --- | --- |
| ONC | 0.37 | 0.35 | a | 0.15 | 40.59 |  |
| 1h | 0.42 | 0.40 | abcd | 0.17 | 39.98 | 13% |
| 2h | 0.40 | 0.39 | abcd | 0.17 | 41.58 | 10% |
| 3h | 0.45 | 0.44 | b | 0.17 | 37.25 | 25% |
| 4h | 0.39 | 0.38 | acd | 0.16 | 39.61 | 9% |
| 5h | 0.39 | 0.38 | acd | 0.14 | 36.63 | 7% |
| 6h | 0.38 | 0.37 | ad | 0.14 | 36.50 | 5% |
| 7h | 0.40 | 0.39 | bcd | 0.14 | 36.29 | 12% |
| 8h | 0.39 | 0.38 | acd | 0.19 | 47.60 | 9% |
| 24h | 0.41 | 0.40 | bc | 0.16 | 38.08 | 14% |

**Table S3: Summary statistics of single-cell dry-mass distributions and OD600 values reached at the time of sampling in *Vibrio natriegens* under chemical stress with copper, zinc, and diclofenac**. Reported metrics include mean dry mass (DM), median DM, DM interquartile range (IQR), DM coefficient of variation (CV), and percentage change in median dry mass, plus average OD600 values (± standard deviation) at time of sampling.

| **Treatment** | **Mean DM** | **Median DM** | **DM IQR** | **DM CV** | **DM skewness** | **median DM change vs. control** | **average OD600 (± SD)** |
| --- | --- | --- | --- | --- | --- | --- | --- |
| **Control** | **0.37** | **0.36** | **0.22** | **39.85** | **0.40** |  | 0.37 (± 0.01) |
| **0.01 mg L^-1^ Cu** | 0.40 | 0.39 | 0.24 | 41.44 | 0.47 | 7% | 0.36 (± 0.01) |
| **0.1 mg L^-1^ Cu** | 0.36 | 0.35 | 0.22 | 41.75 | 0.47 | -4% | 0.33 (± 0.01) |
| **1 mg L^-1^ Cu** | 0.39 | 0.38 | 0.23 | 39.68 | 0.47 | 5% | 0.36 (± 0.01) |
| **10 mg L^-1^ Cu** | 0.35 | 0.34 | 0.23 | 42.01 | 0.48 | -5% | 0.33 (± 0.01) |
| **50 mg L^-1^ Cu** | 0.31 | 0.30 | 0.19 | 40.96 | 0.59 | -15% | 0.23 (± 0.05) |
| **100 mg L^-1^ Cu** | 0.27 | 0.26 | 0.16 | 40.37 | 0.69 | -28% | 0.18 (± 0.05) |
|  |  |  |  |  |  |  |  |
| **0.01 mg L^-1^ Zn** | 0.41 | 0.39 | 0.23 | 38.07 | 0.60 | 9% | 0.37 (± 0.02) |
| **0.1 mg L^-1^ Zn** | 0.40 | 0.38 | 0.21 | 38.25 | 0.60 | 4% | 0.36 (± 0.01) |
| **1 mg L^-1^ Zn** | 0.40 | 0.38 | 0.22 | 37.66 | 0.53 | 6% | 0.36 (± 0.01) |
| **10 mg L^-1^ Zn** | 0.38 | 0.36 | 0.22 | 40.70 | 0.69 | -1% | 0.37 (± 0.01) |
| **50 mg L^-1^ Zn** | 0.37 | 0.35 | 0.21 | 40.86 | 0.70 | -4% | 0.34 (± 0.02) |
| **100 mg L^-1^ Zn** | 0.29 | 0.26 | 0.19 | 47.04 | 1.03 | -28% | 0.22 (± 0.09) |
|  |  |  |  |  |  |  |  |
| **0.01 mg L^-1^ diclofenac** | 0.37 | 0.36 | 0.21 | 38.68 | 0.54 | -1% | 0.37 (± 0.03) |
| **0.1 mg L^-1^ diclofenac** | 0.32 | 0.31 | 0.18 | 40.52 | 0.64 | -14% | 0.35 (± 0.02) |
| **1 mg L^-1^ diclofenac** | 0.37 | 0.36 | 0.21 | 38.61 | 0.48 | 0% | 0.37 (± 0.02) |
| **10 mg L^-1^ diclofenac** | 0.37 | 0.36 | 0.20 | 37.44 | 0.39 | 1% | 0.35 (± 0.02) |
| **50 mg L^-1^ diclofenac** | 0.25 | 0.23 | 0.15 | 41.60 | 0.81 | -37% | 0.23 (± 0.04) |
| **100 mg L^-1^ diclofenac** | 0.24 | 0.23 | 0.14 | 39.86 | 0.79 | -36% | 0.07 (± 0.05) |

**Table S4**: **Summary statistics of single-cell dry-mass distributions and OD600 values reached at the time of sampling in *Vibrio natriegens* under chemical stress with bisphenol A, bisphenol E, and bisphenol Z**. Reported metrics include mean dry mass (DM), median DM, DM interquartile range (IQR), DM coefficient of variation (CV), and percentage change in median dry mass, plus average OD600 values (± standard deviation) at time of sampling.

| **Treatment** | **Mean DM** | **Median DM** | **DM IQR** | **DM CV** | **DM skewness** | **median DM change vs. DMSO** | **average  OD600 (± SD)** |
| --- | --- | --- | --- | --- | --- | --- | --- |
| **Control** | 0.34 | 0.32 | 0.21 | 21.00 | 0.69 |  | 0.37 (± 0.01) |
| **DMSO** | **0.32** | **0.31** | **0.20** | **20.00** | **0.41** |  | 0.35 (± 0.01) |
| **0.001 mg L^-1^ BPA** | 0.33 | 0.32 | 0.20 | 20.00 | 0.36 | 3% | 0.35 (± 0.01) |
| **0.01 mg L^-1^ BPA** | 0.34 | 0.33 | 0.20 | 20.00 | 0.48 | 5% | 0.37 (± 0.03) |
| **0.1 mg L^-1^ BPA** | 0.35 | 0.34 | 0.20 | 20.00 | 0.41 | 9% | 0.36 (± 0.01) |
| **1 mg L^-1^ BPA** | 0.33 | 0.32 | 0.19 | 19.00 | 0.51 | 3% | 0.36 (± 0.01) |
| **10 mg L^-1^ BPA** | 0.33 | 0.33 | 0.21 | 21.00 | 0.31 | 5% | 0.34 (± 0.01) |
|  |  |  |  |  |  |  |  |
| **0.001 mg L^-1^ BPE** | 0.40 | 0.39 | 0.20 | 23.00 | 0.49 | 24% | 0.37 (± 0.01) |
| **0.01 mg L^-1^ BPE** | 0.35 | 0.33 | 0.23 | 21.00 | 0.93 | 7% | 0.37 (± 0.01) |
| **0.1 mg L^-1^ BPE** | 0.36 | 0.34 | 0.21 | 21.00 | 0.61 | 10% | 0.37 (± 0.00) |
| **1 mg L^-1^ BPE** | 0.35 | 0.34 | 0.21 | 20.00 | 0.47 | 8% | 0.37 (± 0.01) |
| **10 mg L^-1^ BPE** | 0.36 | 0.35 | 0.20 | 21.00 | 0.56 | 11% | 0.36 (± 0.01) |
|  |  |  |  |  |  |  |  |
| **0.001 mg L^-1^ BPZ** | 0.38 | 0.36 | 0.20 | 21.00 | 0.54 | 17% | 0.35 (± 0.00) |
| **0.01 mg L^-1^ BPZ** | 0.39 | 0.37 | 0.21 | 22.00 | 0.52 | 19% | 0.36 (± 0.01) |
| **0.1 mg L^-1^ BPZ** | 0.37 | 0.35 | 0.22 | 21.00 | 0.49 | 12% | 0.36 (± 0.01) |
| **1 mg L^-1^ BPZ** | 0.40 | 0.39 | 0.21 | 22.00 | 0.50 | 24% | 0.36 (± 0.01) |
| **10 mg L^-1^ BPZ** | 0.38 | 0.37 | 0.22 | 22.00 | 0.62 | 17% | 0.32 (± 0.01) |

**References**

1.	Lagerström M, Ferreira J, Ytreberg E, Eriksson-Wiklund AK. Flawed risk assessment of antifouling paints leads to exceedance of guideline values in Baltic Sea marinas. Environmental Science and Pollution Research. 2020;27:27674–87. doi:10.1007/s11356-020-08973-0

2.	Zhao G, Liang R, Wang F, Ding J, Qin W. An all-solid-state potentiometric microelectrode for detection of copper in coastal sediment pore water. Sens Actuators B Chem. 2019;279:369–73. doi:10.1016/j.snb.2018.09.125

3.	Judd NL, Toll JE, McPeek K, Baldwin A, Bergquist B, Tobiason K, et al. Collection and use of porewater data from sediment bioassay studies for understanding exposure to bioavailable metals. Integr Environ Assess Manag. 2021;18:1321–34. doi:https://doi.org/10.1002/ieam.4537

4.	Birch GF, Lee JH, Tanner E, Fortune J, Munksgaard N, Whitehead J, et al. Sediment metal enrichment and ecological risk assessment of ten ports and estuaries in the World Harbours Project. Mar Pollut Bull. 2020;155:111129. doi:10.1016/j.marpolbul.2020.111129

5.	Niu Y, Chen F, Li Y, Ren B. Trends and Sources of Heavy Metal Pollution in Global River and Lake Sediments from 1970 to 2018. In: Reviews of Environmental Contamination and Toxicology. Volume 257. Cham, Switzerland: Springer; 2020. p. 1–36. doi:https://doi.org/10.1007/398_2020_59

6.	Li Y, Wang Z, Cai Y, Xiao K, Guo Z, Pan F. High resolution dissolved heavy metals in sediment porewater of a small estuary: Distribution, mobilization and migration. Science of the Total Environment. 2023;905:167238. doi:10.1016/j.scitotenv.2023.167238

7.	Sathishkumar P, Meena RAA, Palanisami T, Ashokkumar V, Palvannan T, Gu FL. Occurrence, interactive effects and ecological risk of diclofenac in environmental compartments and biota - A review. Science of the Total Environment. 2020;698:134057. doi:10.1016/j.scitotenv.2019.134057

8.	Bonnefille B, Gomez E, Courant F, Escande A, Fenet H. Diclofenac in the marine environment: A review of its occurrence and effects. Mar Pollut Bull. 2018;131:496–506. doi:10.1016/j.marpolbul.2018.04.053

9.	Liu J, Zhang L, Lu G, Jiang R, Yan Z, Li Y. Occurrence, toxicity and ecological risk of Bisphenol A analogues in aquatic environment – A review. Ecotoxicol Environ Saf. 2021;208:111481. doi:10.1016/j.ecoenv.2020.111481

10.	Fromme H, Küchler T, Otto T, Pilz K, Müller J, Wenzel A. Occurrence of phthalates and bisphenol A and F in the environment. Water Res. 2002;36:1429–38. doi:https://doi.org/10.1016/S0043-1354(01)00367-0

11.	Caban M, Stepnowski P. The quantification of bisphenols and their analogues in wastewaters and surface water by an improved solid-phase extraction gas chromatography/mass spectrometry method. Environmental Science and Pollution Research. 2020;27:28829–39. doi:10.1007/s11356-020-09123-2

12.	Russo G, Laneri S, Di Lorenzo R, Ferrara L, Grumetto L. The occurrence of selected endocrine-disrupting chemicals in water and sediments from an urban lagoon in Southern Italy. Water Environment Research. 2021;93:1944–58. doi:10.1002/wer.1566
